# Metastasizing Pleomorphic Adenoma: A Systematic Review and Pooled Case-Report Analysis

**DOI:** 10.3390/cancers18142345

**Published:** 2026-07-20

**Authors:** Stefan Janik, Blazen Marijic, Filip Tudor, Katharina B. Erovic, Boban M. Erovic

**Affiliations:** 1 Institute of Head and Neck Diseases, Evangelical Hospital, 1180 Vienna, Austria; mail@hno-janik.at (S.J.); blazen.marijic@uniri.hr (B.M.); 2 Department of Otorhinolaryngology, University Hospital Wiener Neustadt, 2700 Wiener Neustadt, Austria; 3 Department of Otorhinolaryngology, Head and Neck Surgery, Clinical Hospital Center Rijeka, 51000 Rijeka, Croatia; filip.tudor@medri.uniri.hr; 4 Faculty of Psychology, Sigmund Freud Private University, Freudplatz 1, 1020 Vienna, Austria; 62401544@mail.sfu.ac.at

**Keywords:** metastasizing pleomorphic adenoma, pleomorphic adenoma, salivary gland neoplasm, parotid gland, systematic review, metastasis, surgery, overall survival

## Abstract

**Background/Objectives:** Metastasizing pleomorphic adenoma (MPA) is a rare salivary gland tumor characterized by benign pleomorphic adenoma morphology despite regional or distant metastatic behavior. Because most evidence derives from case reports and small series, this systematic review and pooled case-report analysis aimed to summarize published patient-level data and to explore clinicopathologic features, metastatic distribution, treatment, and survival without inferring causal treatment effects. **Methods:** PubMed, Scopus, Google Scholar, and reference lists were searched for English-language reports published up to 1 August 2025. Eligible reports described primary pleomorphic adenoma and metastatic disease reported as MPA with benign morphology; carcinoma ex pleomorphic adenoma and other malignant pleomorphic adenoma variants were excluded. The primary pooled analysis was restricted to 122 published case-report patients. Institutional cases are presented separately as illustrative cases and were not included in the primary pooled statistical analyses. Unknown, not reported, NA, and unclear values were treated as missing, and denominators are reported. Because only 16 deaths were available, survival analyses were exploratory and no multivariable Cox model was fitted. No formal risk-of-bias or certainty-of-evidence assessment was performed. **Results**: In the 122 published patients, sex was available for 121 patients and 71/121 (58.7%) were female. Primary tumors most frequently arose in the parotid gland (92/122, 75.4%), followed by the submandibular gland (15/122, 12.3%) and palate/soft palate (10/122, 8.2%). The median interval between primary pleomorphic adenoma and MPA was 12 years (IQR 7–21; range 0–69; *n* = 118). Metastatic site was reported in 120 patients and was non-mutually exclusive: bone/skeleton and lymph nodes/neck were each reported in 42/120 patients (35.0%), followed by lung/pulmonary metastases in 31/120 (25.8%). Treatment information was available for 98/122 patients. MPA-directed surgery was performed in 86/98 patients with available treatment information (87.75%). The non-surgical cohort received radiotherapy, chemotherapy, palliative treatment, treatment refusal, observation, or other non-surgical strategies. A total of 72 patients had complete covariate data for the reported Cox analyses, including 16 deaths. Estimated 1- and 5-year overall survival were 89.97% and 66.2%, respectively. Surgery was associated with improved overall survival in exploratory Kaplan–Meier comparison (log-rank *p* = 0.008) and univariable Cox analysis (HR 0.119, 95% CI 0.018–0.810, *p* = 0.030), but this association should not be interpreted as causal. **Conclusions**: MPA remains difficult to predict and the available evidence is limited by case-report design, missing data, publication bias, and heterogeneous follow-up. Surgical management may be considered in selected patients when technically feasible and clinically appropriate, but the observed survival association is exploratory and may be influenced by selection bias and disease characteristics. Multicenter registries, standardized reporting, centralized pathology review, molecular characterization, and longer follow-up are needed.

## Simple Summary

Metastasizing pleomorphic adenoma (MPA) is a very rare salivary gland tumor that appears benign under the microscope but can metastasize to regional or distant sites. Because the available evidence consists predominantly of case reports and small case series, conclusions regarding prognosis and treatment are low-certainty and hypothesis-generating. This systematic review and pooled case-report analysis summarizes 122 published patients with MPA, with explicit reporting of denominators and missing data. Metastatic lesions most often involved bone, lymph nodes/neck, and lung. Surgical management was associated with improved survival in exploratory univariable analyses; however, this association cannot be interpreted as an independent causal treatment effect because of selection bias, resectability, disease burden, performance status, and reporting completeness. Long-term clinical awareness after pleomorphic adenoma, especially recurrent disease, remains warranted, and surgery may be considered when technically feasible and clinically appropriate.

## 1. Introduction

Pleomorphic adenoma (PA) is the most common benign salivary gland tumor and arises most frequently in the parotid gland [1]. Although PA is generally considered indolent, its clinical behavior may be unpredictable. Recurrent pleomorphic adenoma (rPA) can develop after incomplete excision or tumor spillage and often presents as multifocal nodules, thereby complicating subsequent surgical management [2]. In rare cases, long-standing or recurrent PA may undergo malignant transformation to carcinoma ex pleomorphic adenoma, an aggressive tumor associated with poor prognosis [3]. Histologically, PA is classically characterized by a biphasic epithelial and myoepithelial component embedded in a variable chondromyxoid, myxoid, or hyalinized stromal background. Because of this architectural and cytologic diversity, PA has historically also been referred to as a “mixed tumor” [4,5]. In routine clinical practice, PA usually presents as a painless, slowly enlarging, well-circumscribed mass, most often in the superficial lobe of the parotid gland. Nevertheless, despite its benign histologic appearance, PA is not always biologically innocuous. Its clinical course may be influenced by tumor size, duration, anatomic site, completeness of excision, capsular integrity, pseudopod-like extensions, satellite nodules, and the presence of previous surgical manipulation.

The standard treatment of PA is complete surgical excision with adequate preservation of facial nerve function and surrounding structures. In the parotid gland, surgical approaches have evolved from simple enucleation toward superficial parotidectomy, extracapsular dissection, partial superficial parotidectomy, or total parotidectomy depending on tumor location, size, and surgeon experience [6]. This evolution reflects the recognition that incomplete removal, capsular violation, tumor spillage, and residual microscopic disease may predispose to recurrence. Such multifocal recurrence is clinically important because it complicates revision surgery, increases the risk of facial nerve injury, and may require more aggressive treatment strategies, including postoperative radiotherapy in selected patients.

Metastasizing pleomorphic adenoma (MPA) represents an unusual and clinically important entity. In contrast to carcinoma ex pleomorphic adenoma, MPA retains the benign histologic features of PA or rPA but is capable of regional or distant spread, most commonly to lymph nodes, bone, and lung [1,7,8,9]. This combination of benign morphology and metastatic behavior creates a diagnostic and therapeutic paradox. Current treatment is primarily directed toward complete removal of the primary or recurrent tumor and metastases whenever technically and clinically feasible. MPA has also been described in kidney, skin, liver, central nervous system, and other soft tissue sites [1,7,8,9,10]. Lymphatic spread may manifest as cervical nodal disease in the setting of a parotid or other head and neck primary, whereas hematogenous spread may result in skeletal, pulmonary, or visceral metastases. In many cases, metastatic disease is detected years or decades after initial PA treatment, and the relationship between the original benign tumor and later metastatic deposits may only become apparent after careful review of the patient’s clinical history and pathology. Because the metastatic lesions can be histologically bland, they may be misinterpreted as benign ectopic salivary gland tissue, myoepithelial lesions, or other low-grade tumors if the previous diagnosis of PA is not known [11]. The pathogenesis of MPA remains poorly understood. Recurrence, repeated surgical manipulation, incomplete excision, and tumor spillage have been proposed as risk factors; however, de novo presentations and emerging molecular data suggest that biological factors may also contribute to metastatic behavior [1,7,8,9,12]. Because MPA is exceedingly rare, evidence is limited to case reports and small series. The aim of this systematic review and pooled analysis was therefore to summarize current knowledge, analyze potential prognostic factors, and evaluate treatment strategies in relation to outcomes in patients with MPA.

Several mechanisms have been proposed to explain the metastatic capacity of an otherwise histologically benign neoplasm. One hypothesis emphasizes iatrogenic dissemination. Tumor rupture, incomplete excision, enucleation, repeated surgery, or incisional biopsy may theoretically allow tumor cells to enter lymphatic or vascular channels or seed the operative field. This concept is supported by the frequent association of MPA with rPA and repeated surgical interventions [2,13,14]. However, iatrogenic spread alone does not fully explain the entity. MPA has also been reported in patients without a documented history of recurrent disease or extensive surgical manipulation [15,16,17,18]. Moreover, not all patients with multiple recurrences develop metastases, and many recurrent PAs remain confined to the original surgical field. These observations suggest that tumor-intrinsic biological factors may contribute to metastatic potential [19].

Emerging molecular findings support this possibility. Rearrangements involving PLAG1 and HMGA2 are well-recognized drivers in PA, and recurrent PLAG1/HMGA2 alterations have also been documented in MPA [12]. The identification of a novel HMGA2-TMTC2 fusion in MPA suggests that at least some cases may harbor distinctive molecular events associated with dissemination [12]. In addition, biomarkers linked to angiogenesis and metastatic behavior, such as CD105/endoglin, have been investigated in PA, rPA, and MPA [20,21]. These findings remain preliminary, but they indicate that MPA may not simply represent accidental displacement of otherwise conventional PA cells. Instead, it may reflect a rare biological subset of PA in which specific molecular alterations, stromal interactions, angiogenic changes, or host factors permit survival and growth at distant sites.

The rarity of MPA has limited systematic evaluation. Most available evidence consists of individual case reports, small institutional series, and narrative or systematic reviews that combine heterogeneous cases from different eras. As a result, several clinically relevant questions remain unresolved. It is unclear which patients with PA or rPA are at greatest risk of developing MPA, whether specific metastatic sites are associated with age, sex, recurrence history, or latency, and whether treatment of metastatic disease improves survival. Surgical resection is generally recommended when feasible, particularly for isolated regional or distant lesions, but evidence supporting this approach is largely derived from retrospective observations. The roles of radiotherapy, chemotherapy, systemic therapy, and surveillance imaging remain poorly defined [22]. Against this background, a pooled analysis of published and institutional cases may help clarify patterns of presentation, metastatic distribution, treatment, and outcome. Although such an approach cannot eliminate the limitations inherent to retrospective case-based literature, it can identify recurring clinicopathologic features and generate hypotheses for future multicenter studies.

Compared with prior systematic reviews, the present revision emphasizes the case-report nature of the evidence and updates the analysis as a pooled case-report dataset rather than a conventional treatment-effect meta-analysis. The main added value is transparent patient-level tabulation of published cases, explicit non-missing denominators for each variable, revised missing-data handling, and sparse-data-aware exploratory survival modelling. The analysis is intended to identify recurring clinical patterns and generate hypotheses for registry-based studies, not to establish independent treatment effects.

## 2. Materials and Methods

### 2.1. Search Strategy

A comprehensive literature search was performed in PubMed, Scopus, and Google Scholar for English-language reports published up to 1 August 2025. The PubMed strategy was: (“metastasizing pleomorphic adenoma” OR “metastasising pleomorphic adenoma” OR “benign metastasizing pleomorphic adenoma” OR (“pleomorphic adenoma” AND (metastasis OR metastatic OR metastases))). The Scopus strategy was: TITLE-ABS-KEY (“metastasizing pleomorphic adenoma” OR “metastasising pleomorphic adenoma” OR “benign metastasizing pleomorphic adenoma” OR (“pleomorphic adenoma” AND (metastasis OR metastatic OR metastases))). Google Scholar was searched using the phrases “metastasizing pleomorphic adenoma”, “metastasising pleomorphic adenoma”, “benign metastasizing pleomorphic adenoma”, and “pleomorphic adenoma metastasis”; results were screened in relevance order until no additional potentially eligible cases were identified. All reference-list-derived records were already captured in the database set. Because Google Scholar searches are difficult to reproduce and the exact number of screened Google Scholar records was not documented prospectively, this limitation is acknowledged explicitly.

**Figure 1:**
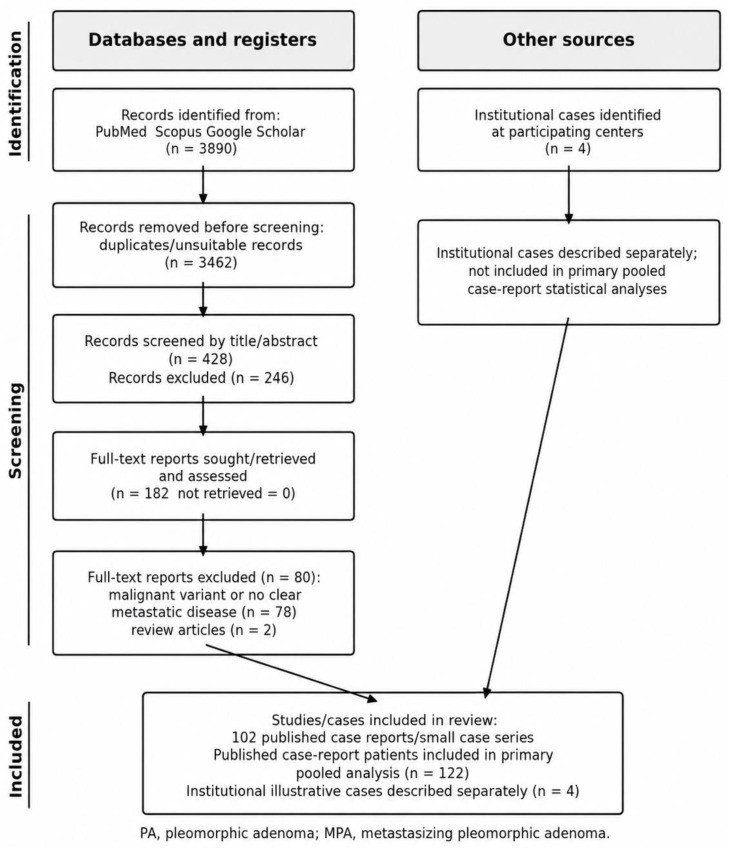
PRISMA 2020 flow diagram of study selection. The primary pooled case-report analysis included 122 published patients from 102 reports; four institutional cases are described separately and were not included in the primary pooled statistical tests.

This systematic review and pooled case-report analysis was reported in accordance with the PRISMA 2020 statement [23]. A Supplementary List Table S3 of the 80 excluded full-text reports with individual reasons is included as Supplementary Material. The study selection process is summarized in Figure 1. The review protocol was not preregistered, no registration number is available, and no separate review protocol was prepared; this is a methodological limitation because it may increase susceptibility to post hoc review decisions. No formal certainty-of-evidence assessment was undertaken because the evidence base consisted almost entirely of case reports and small series, but reporting limitations and missing data are summarized and discussed. B.M., F.T. and S.J. participated in the literature search, and independently reviewed and read the literature. Each stage was conducted independently.

### 2.2. Inclusion and Exclusion Criteria

Studies were included if they fulfilled the following criteria: (1) reported diagnosis of a primary pleomorphic adenoma; (2) reported regional or distant metastatic disease diagnosed as MPA; (3) benign pleomorphic adenoma morphology reported in the metastatic lesion; (4) availability of extractable patient-level clinical data; and (5) publication in English. Editorials, meeting abstracts, review articles without extractable original cases, cases with carcinoma ex pleomorphic adenoma or another malignant pleomorphic adenoma variant, and reports describing only recurrent benign lesions without clear evidence of metastasis were excluded. Published pathological diagnoses were accepted as reported; central pathology review, immunohistochemistry, and molecular confirmation were not uniformly available and were not required for inclusion. This limitation is important because occult malignant transformation cannot be excluded with certainty in all historical cases.

### 2.3. Study Selection and Data Extraction

Database searches identified 3890 records. After removal of duplicates or otherwise unsuitable records before screening (*n* = 3462), 428 records were screened by title and abstract, and 246 records were excluded. Full-text reports were sought and assessed for 182 records; no reports were unavailable. Eighty full-text reports were excluded, including 78 because metastasis arose from a malignant variant of pleomorphic adenoma or because the lesion represented recurrence without clear metastatic criteria, and two because they were review articles. Finally, 102 case reports or small case series comprising 122 published patients were included in the primary pooled case-report analysis. Four institutional cases from the participating centers are presented separately as illustrative cases and were not included in the primary pooled statistical tests or survival models. Cases treated between 2009 and 2021 were identified from clinical charts. Patients were included consecutively according to predefined institutional eligibility criteria at Departments of Vienna and Rijeka. Cases were excluded for incomplete records, insufficient follow-up, or lack of diagnostic confirmation.

Screening, full-text assessment, and data extraction were performed using a standardized extraction spreadsheet; disagreements were resolved by consensus and no automation tools were used for screening or extraction. Reviewer initials are B.M., S.J, and F.T.

### 2.4. Variables and Outcomes

Extracted variables included patient age and sex; primary PA location, tumor size, multifocality, clinical presentation, diagnostic work-up, cytopathologic assessment, treatment approach, intraoperative tumor rupture, and surgical margin status. Outcomes related to PA included local recurrence, multiple recurrences, time to recurrence, and overall treatment outcome. For MPA, extracted variables included age at MPA diagnosis, interval between PA and MPA diagnosis, metastatic site, number and size of metastatic lesions, radiologic evaluation (computed tomography, magnetic resonance imaging, ultrasound, positron emission tomography–computed tomography, and X-ray), treatment strategy, follow-up, and MPA recurrence.

### 2.5. Statistical Analysis

All analyses were exploratory and were calculated using IBM SPSS Statistics Version 30.0.0.0 (172). Categorical variables were summarized as *n*/*N* (%) using the number of cases with available information as the denominator. Continuous variables were summarized as median, interquartile range (IQR), range, and available *n*. Values reported as unknown, not reported, NA, or unclear were treated as missing and were not imputed. Metastatic localization was treated as a non-mutually exclusive categorical variable; therefore, Pearson or Spearman correlation was not used to analyze localization as an ordinal continuous variable. Overall survival was defined as the interval from MPA diagnosis to death from any cause or last known follow-up. Kaplan–Meier estimates and log-rank tests were used descriptively. Univariable Cox models are reported with *N*, number of events, HRs, 95% CIs, and *p* values. Given only 16 deaths in the usable overall-survival dataset, no multivariable Cox regression model was fitted. No multiplicity adjustment was applied, and all *p* values should be interpreted as hypothesis-generating rather than confirmatory.

## 3. Results

### 3.1. Study Cohort

**Figure 2:**
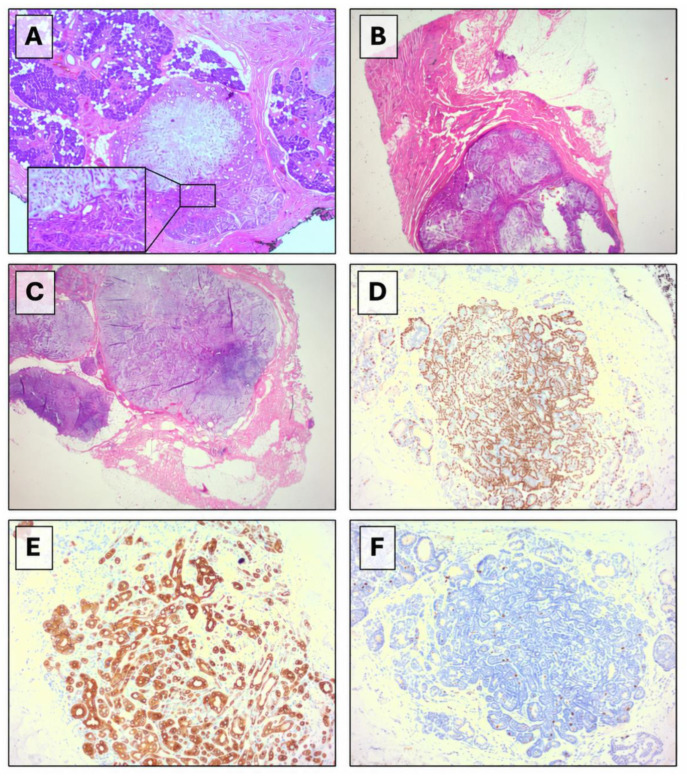
Metastasizing pleomorphic adenoma (Case 4). The primary pleomorphic adenoma of the parotid gland is shown for a female patient who underwent initial surgery at 16 years of age (**A**). Localized (**B**) and diffuse (**C**) metastases of pleomorphic adenoma occurred in the head and neck region 12 years after initial treatment. Immunohistochemical staining for p63 (**D**), CK7 (**E**), and Ki-67 (**F**) is shown for the metastatic lesions. Strong expression of p63 and CK7 indicate a prominent epithelial phenotype with evidence of basal, squamous, or myoepithelial differentiation. In contrast, Ki-67 expression is very weak, suggesting a low proliferative index and limited cellular turnover within the examined lesion. The four institutional cases are summarized separately in Table 2; Case 4 is illustrated in Figure 2. The overview magnification is ×40 and the detail view is magnified ×100.

A total of 122 published case-report patients from 102 reports were included in the primary pooled case-report analysis (Table 1). The four institutional cases are summarized separately in Table 2; Case 4 is illustrated in Figure 2. Histopathological examination of Case 4 demonstrated pleomorphic adenoma morphology in the metastatic deposits. Immunohistochemical findings for p63, CK7, and Ki-67 are illustrated in Figure 2D–F. None of the institutional cases were incorporated into the primary pooled statistical tests or survival models. Among published cases, sex was available for 121 patients: 71/121 (58.7%) were female and 50/121 (41.3%) were male. Most cases were published between 2000 and 2025, although the first reported MPA case was published in 1942.

Most primary tumors originated in the parotid gland (92/122, 75.4%), followed by the submandibular gland (15/122, 12.3%) and palate/soft palate (10/122, 8.2%). Less common primary sites included the nasal septum or nasal cavity (2/122, 1.6%), tongue (1/122, 0.8%), lip (1/122, 0.8%), and other or unclear sites (1/122, 0.8%).

A history of primary PA recurrence was available for 116 patients and was present in 80/116 (69.0%) (Table 1). Multiple primary PA recurrences were reported in 46/116 patients (39.7%). The number of recurrences was numerically available for 70 patients, with a median of 2 recurrences (IQR 1–2; range 0–11). These estimates should be interpreted cautiously because recurrence details were incompletely reported in many historical reports.

### 3.2. Characteristics and Distribution of MPA

**Figure 3:**
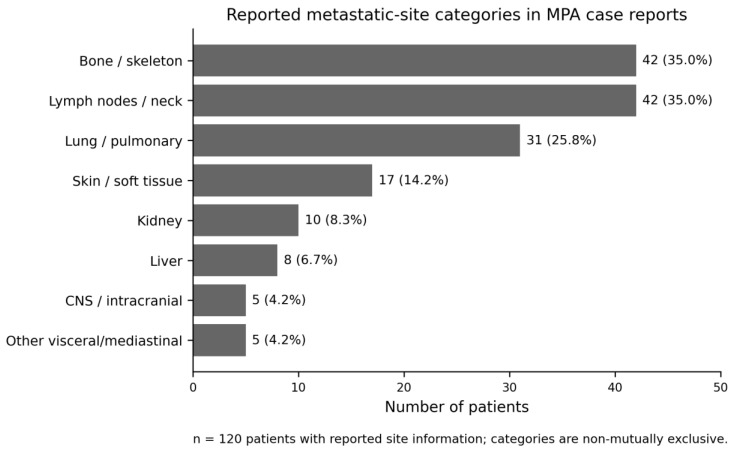
Descriptive distribution of reported metastatic-site categories in patients with metastasizing pleomorphic adenoma (MPA). Site categories are non-mutually exclusive; statistical inference does not treat localization as an ordinal continuous variable.

The interval between primary PA and MPA was available for 118 patients and had a median of 12 years (IQR 7–21; range 0–69 years). Among 71 patients with available post-treatment recurrence information, 18/71 (25.4%) experienced MPA recurrence. Metastatic site was reported in 120/122 patients. Because many patients had more than one site involved, site categories were treated as non-mutually exclusive. Bone/skeleton and lymph nodes/neck were each reported in 42/120 patients (35.0%), followed by lung/pulmonary metastases in 31/120 (25.8%), skin or soft tissue in 17/120 (14.2%), kidney in 10/120 (8.3%), liver in 8/120 (6.7%), central nervous system/intracranial sites in 5/120 (4.2%), and other visceral or mediastinal sites in 5/120 (4.2%) (Figure 3). Multiple metastatic lesions were reported in 69/113 patients with available information (61.1%).

### 3.3. Association Between Age and MPA Localization

In the analysis, metastatic localization was treated as a non-mutually exclusive categorical variable rather than as an ordinal variable. Accordingly, no Pearson or Spearman correlation between patient age and MPA localization was calculated. Apparent differences in age distribution by metastatic site are considered descriptive and hypothesis-generating only, because they may reflect reporting patterns, surveillance intensity, disease duration, and treatment selection.

### 3.4. Treatment of MPA

Treatment information was available for 98/122 patients. MPA-directed surgery was performed in 86/98 patients with available treatment information (87.75%), whereas 12/98 (12.2%) were managed without MPA-directed surgery. Treatment information was missing for 24/122 patients. In particular, for 24 patients no information regarding treatment was available, whereas 12 other patients had non-surgical, palliative, observation, refusal, inoperable, radiotherapy, or chemotherapy. Because patients selected for surgery are likely to differ systematically in disease burden, resectability, site of metastasis, performance status, and reporting completeness, treatment comparisons were interpreted as exploratory associations rather than independent treatment effects.

### 3.5. Outcomes and Prognostic Analyses

Vital status was available for 85/122 patients; 69/85 (81.2%) were alive and 16/85 (18.8%) were dead at last follow-up. Overall-survival analysis was possible for 72 patients with both numeric follow-up and known vital status, including 16 deaths. Estimated 1-year and 5-year overall survival were 89.97% and 66.2%, respectively.

**Figure 4:**
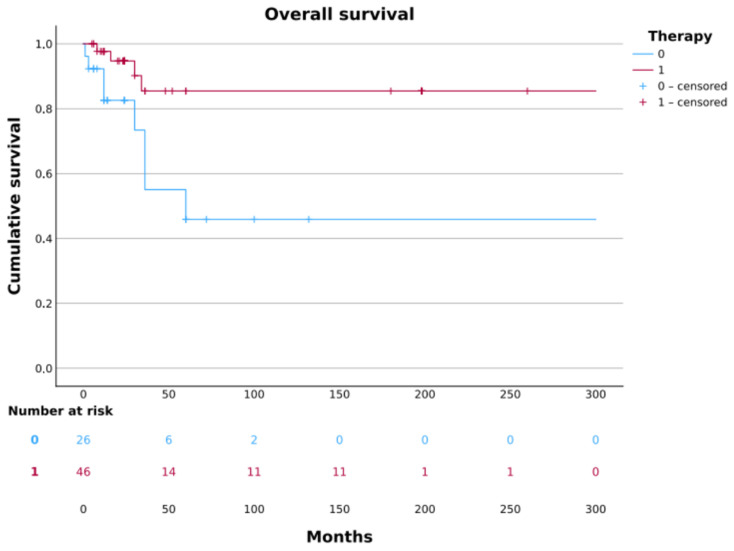
Exploratory overall survival according to MPA-directed surgery in patients with known surgery status, numeric follow-up, and known vital status. This comparison is hypothesis-generating and likely confounded by indication (log-rank: *p* = 0.008). MPA surgery = 1; other/no MPA-directed surgery = 0, *n* = 72, 16 deaths.

An exploratory univariable Cox proportional hazards model is summarized in Table 3. MPA-directed surgery was associated with improved overall survival in the exploratory univariable Cox model (HR 0.119, 95% CI 0.018–0.810, *p* = 0.030; Table 3). The corresponding Kaplan–Meier comparison yielded a log-rank *p* = 0.008 (Figure 4). The dichotomized primary-site category (salivary glands vs. other) also showed an exploratory association with overall survival (HR 0.131, 95% CI 0.017–0.981, *p* = 0.048). Age, modelled continuously per one-year increase, was not significantly associated with survival (HR 1.02, 95% CI 0.98–1.07, *p* = 0.321). Likewise, sex (HR 0.85, 95% CI 0.21–3.40, *p* = 0.815), the presence of lung and/or bone metastases (HR 0.91, 95% CI 0.35–2.38, *p* = 0.842), and multiple lesions (HR 2.35, 95% CI 0.40–13.80, *p* = 0.343) were not significantly associated with overall survival. Given the limited number of events and the resulting imprecision of several estimates, these findings should be interpreted as exploratory, and the observed association between therapy and survival should not be interpreted as an independent causal treatment effect.

## 4. Discussion

This systematic review and pooled case-report analysis included 122 published patients with MPA. Four institutional cases are presented separately as illustrative cases and were not included in the primary pooled statistical tests. Although MPA displays benign histologic features, it can spread regionally or distantly and is generally regarded as a low-grade malignant salivary gland neoplasm. Because the evidence consists predominantly of case reports and small series, all survival findings should be considered low-certainty and hypothesis-generating.

The entity occupies a distinctive position within salivary gland pathology because it contradicts the conventional assumption that histologically benign PA lacks metastatic potential. In clinical terms, MPA behaves neither like ordinary PA nor like conventional high-grade salivary carcinoma [11]. Its course is often prolonged, latency may extend over many years, and metastatic deposits may remain histologically bland. Nevertheless, the development of regional or distant disease can lead to repeated interventions, functional morbidity, and disease-related mortality.

Overall, the published-case dataset showed a modest female predominance among patients with reported sex (58.7%). MPA often occurred many years after initial PA treatment, with a median interval of 12 years between PA and MPA diagnosis. The four institutional cases were all female, developed locoregional head and neck disease, and were alive without recurrence during follow-up; because they derive from a different source of evidence, they are described separately and not combined with the published cases in the revised pooled analyses.

The present cohort confirms that MPA is rare, delayed, and heterogeneous. The long latency is clinically important because patients and clinicians may consider a previously treated benign PA cured after several disease-free years, particularly if the original surgery was performed decades earlier. However, the prolonged interval observed in this and previous analyses indicates that late recurrence or metastasis remains possible, especially in patients with recurrent, multifocal, or incompletely excised tumors [1,2,7,8]. Long-term awareness is therefore essential, even though the absolute risk of MPA is very low.

The demographic profile of the cohort is broadly consistent with the epidemiology of PA, which often affects adults in middle age and shows a slight female predominance in many series. However, MPA should not be viewed as limited to one demographic group. Cases have been reported across a wide age range, and metastatic disease may occur in both younger and older patients. The favorable outcome of the four institutional cases likely reflects the predominance of surgically accessible regional disease, but the small number of cases prevents firm conclusions. The short-latency presentation in one institutional patient illustrates that MPA cannot always be explained by decades of recurrent disease.

A history of recurrent PA was common in the pooled cohort, supporting the established association between recurrence and later metastatic behavior. Recurrent PA is often multifocal, particularly after previous enucleation, capsular rupture, or tumor spillage. Small nodules may be distributed throughout scarred tissue planes, making complete eradication difficult and increasing the likelihood of repeated procedures. Each additional operation may increase surgical complexity and may theoretically increase the opportunity for lymphovascular access or mechanical dissemination. This finding should be interpreted cautiously because recurrence data were incompletely reported and because the analysis included only patients who had already developed MPA. Still, it suggests that recurrence burden alone is insufficient to explain metastatic risk.

In the published-case cohort, metastatic-site categories were non-mutually exclusive. Bone/skeleton and lymph nodes/neck were the most frequently reported categories, each present in 35.0% of patients with available site data, followed by lung/pulmonary metastases in 25.8%. Differences from prior reviews may reflect different case definitions, classification of regional disease, duplicate case reporting, missing data, and changes in imaging and reporting practices over time rather than true epidemiologic differences.

Several additional factors may account for the observed differences in metastatic site distribution. First, definitions of regional disease vary across studies. Some authors classify cervical lymph node involvement separately, whereas others combine all head and neck metastases into a single category. Second, older case reports may preferentially report unusual distant metastases, such as skeletal or pulmonary deposits, whereas regional nodal disease may have been underreported or interpreted differently. Third, diagnostic imaging has changed substantially over time, potentially influencing detection of small regional or distant lesions. Finally, because MPA is rare, small differences in inclusion criteria can meaningfully alter the apparent frequency of metastatic sites.

Descriptive age patterns by metastatic localization may have several possible explanations; however, no formal association analysis was performed [24,25]. Younger patients may be more likely to undergo intensive local and regional surveillance after recurrent PA, leading to earlier identification of cervical or locoregional metastases. Alternatively, regional disease in younger patients may reflect local lymphatic dissemination related to prior surgery or tumor multifocality, whereas distant skeletal involvement in older patients may represent a later phase of disease evolution. It is also possible that age-related differences in tumor biology, immune surveillance, vascular environment, or duration of occult disease influence the eventual metastatic pattern. These possibilities remain speculative but warrant further investigation.

The distinction between regional and distant MPA has practical implications. Head and neck metastases, particularly cervical nodal disease or soft tissue deposits, are often surgically accessible and may be treated with curative intent. In contrast, bone, lung, kidney, and multisite disease may be less amenable to complete resection, particularly when lesions are multiple or involve critical structures. This difference may have influenced treatment selection in the present cohort. Therefore, metastatic location is not only a descriptive variable but also a determinant of therapeutic feasibility and survival.

The mechanisms underlying metastatic spread in PA remain unresolved. One hypothesis is that surgical manipulation, tumor rupture, incisional biopsy, or incomplete excision may facilitate hematogenous or lymphatic dissemination [2,13,14]. This hypothesis is supported by the frequent history of recurrence and multiple operations in patients with MPA. However, de novo presentations [15,16,17,18] and the absence of a clear increase in reported MPA cases despite increasing parotidectomy rates [6,26] suggest that iatrogenic dissemination alone cannot explain all cases.

The traditional iatrogenic dissemination hypothesis remains plausible in many cases. PA can have an incomplete or delicate capsule, microscopic extensions, satellite nodules, and close relationships with facial nerve branches. During surgery, particularly older enucleation procedures or revision operations, capsular violation may release tumor cells into the surgical field. Tumor cells could theoretically implant locally, enter lymphatic channels, or gain access to venous circulation. The frequent occurrence of rPA before MPA supports this concept, and the emphasis on careful intraoperative handling, avoidance of rupture, and complete excision remains justified [2,27]. For this reason, prevention of recurrence at the time of initial PA surgery may be the most important practical measure to reduce later morbidity.

However, several observations challenge a purely mechanical explanation. Many patients undergo tumor spillage or develop rPA without ever developing metastasis. In addition, the metastatic lesions in MPA are capable not only of displacement but also of survival, vascularization, and growth at distant sites. These processes require biological properties that go beyond passive implantation. Tumor cells must resist anoikis, adapt to foreign microenvironments, evade immune destruction, and establish stromal and vascular support. Therefore, MPA likely reflects an interaction between surgical or anatomic factors and intrinsic tumor biology. In some patients, previous surgery may provide the opportunity for dissemination; in others, molecular alterations may create a tumor phenotype with genuine metastatic competence.

An alternative hypothesis is that MPA represents a biologically distinct subtype of PA with molecular features that enable metastatic spread. Wasserman et al. reported recurrent PLAG1/HMGA2 rearrangements and identified a novel HMGA2-TMTC2 fusion in MPA [20]. TMTC family alterations and related pathways have been implicated in several malignancies [28,29,30]. Moreover, CD105/endoglin has been proposed as a potential biomarker to distinguish recurrent or metastasizing PA from conventional PA [21]. Endoglin is an endothelial transmembrane glycoprotein implicated in angiogenesis and metastatic progression, and increased CD105 expression has been associated with metastatic behavior in salivary gland neoplasms and other cancers [20,31]. These observations support the need for molecular profiling of PA, rPA, and MPA to identify high-risk lesions.

Molecular studies are still limited but increasingly relevant. PLAG1 and HMGA2 rearrangements are central events in many PAs, and their recurrence in MPA supports a clonal relationship between primary and metastatic lesions [12]. The identification of HMGA2-TMTC2 fusion in MPA is particularly noteworthy because it suggests that rare fusion events may contribute to unusual biological behavior [12]. Although these data do not yet establish causality, they provide a rationale for systematic molecular profiling of PA, rPA, and MPA. Such profiling could clarify whether MPA arises from conventional PA through accumulation of additional alterations, whether it represents a distinct molecular subgroup from the outset, or whether both pathways exist.

Angiogenesis may also play a role in metastatic potential. CD105/endoglin is expressed in proliferating endothelial cells and has been used as a marker of tumor angiogenesis. Prior studies have evaluated CD105 in salivary gland neoplasms and suggested associations with metastatic behavior [20]. Yu et al. proposed that a CD105 ligand identified by phage display may help distinguish recurrent or metastasizing PA from conventional PA [21]. In other malignancies, including renal cell carcinoma, CD105 has been linked to immunosuppression, angiogenesis, and metastasis [31]. These findings are not yet sufficient to establish CD105 as a clinical biomarker for MPA, but they support further study of angiogenic and microenvironmental factors. Future research should integrate histologic assessment, immunohistochemistry, genomic profiling, and clinical outcome data in order to identify reproducible markers of risk.

Despite these emerging hypotheses, MPA pathogenesis remains incompletely explained. Important unanswered questions include why only selected PAs metastasize, which molecular alterations distinguish MPA from conventional PA or carcinoma ex PA, and whether patient age or sex influences metastatic distribution. Given the long latency between PA treatment and MPA detection, long-term surveillance is warranted, particularly after recurrent disease, incomplete excision, or multifocal growth.

The diagnostic work-up of suspected MPA requires careful clinicopathologic correlation. Histologically, metastatic lesions may resemble ordinary PA and lack overt malignant features. This can create diagnostic uncertainty, particularly when the metastatic deposit occurs in an unusual site or when the original PA was treated many years earlier. Pathologists should consider MPA when a benign-appearing epithelial–myoepithelial or chondromyxoid lesion is identified in a lymph node, bone, lung, kidney, or other distant site, especially in a patient with a history of PA or rPA. Conversely, it is essential to exclude carcinoma ex pleomorphic adenoma and other malignant salivary tumors, because management and prognosis differ substantially. Central pathology review, comparison with the original tumor, and molecular testing for shared PLAG1 or HMGA2 alterations may be helpful in selected cases.

Treatment options for MPA are not standardized. In the revised analysis, surgery was associated with better survival in exploratory univariable analysis, but this finding is highly susceptible to confounding by indication. Patients undergoing surgery are likely to have more resectable disease, lower disease burden, better performance status, more localized metastases, and more complete reporting. Therefore, the observed association supports the clinical reasonableness of considering surgery in selected patients, but it does not prove that surgery independently improves survival.

From a therapeutic perspective, complete resection may be considered when technically feasible and clinically appropriate, particularly for isolated regional or surgically accessible metastatic lesions. The role of radiotherapy, chemotherapy, chemoradiotherapy, and systemic therapy remains undefined because available data are sparse and inconsistently reported. Treatment decisions should therefore be individualized according to resectability, symptoms, disease burden, patient fitness, expected morbidity, and patient preference.

Surveillance after PA and rPA is another important issue. Because MPA may occur after long latency, follow-up strategies should be individualized. Routine lifelong imaging for all patients with completely excised conventional PA is unlikely to be practical or cost-effective given the rarity of MPA. However, prolonged clinical follow-up appears reasonable for patients with rPA, multifocal disease, positive or uncertain margins, tumor spillage, or repeated surgery. New neck masses, bone pain, pulmonary symptoms, or unexplained lesions in patients with a history of PA should prompt evaluation with appropriate imaging and tissue diagnosis [32]. Patient education is also important: individuals with rPA should be informed that late recurrence can occur and that new symptoms should not be dismissed solely because the original tumor was benign.

This study has several important limitations. Data were collected retrospectively from case reports and small series, which are inherently prone to publication bias, reporting bias, language restriction, incomplete documentation, inconsistent definitions, and heterogeneous follow-up. Many historical reports lacked complete information on tumor rupture, margins, recurrence history, treatment intent, systemic therapy, imaging, pathology review, immunohistochemistry, molecular testing, and outcomes. Missing data were frequent for follow-up, vital status, treatment, and recurrence variables, and no imputation was performed. There was no centralized pathology review, so occult malignant transformation cannot be definitively excluded in every published case. Duplicate reporting across historical case reports cannot be completely ruled out. No formal certainty-of-evidence grading was performed, and sparse event numbers precluded multivariable survival modelling. Moreover, the absence of a structured appraisal prevents systematic evaluation of case-level diagnostic reliability. Consequently, the results should be interpreted as descriptive and hypothesis-generating only.

The present analysis highlights the need for improved reporting standards. Future reports should provide patient-level data on primary PA site, recurrence history, number of procedures, tumor rupture, margins, metastatic sites, number of lesions, pathology comparison with the primary tumor, immunohistochemistry or molecular findings when available, treatment intent, follow-up duration, and vital status. Standardized reporting would allow for more reliable denominator-based summaries and reduce the instability of pooled analyses in this rare entity.

Rare or unusual metastatic sites may be more likely to be published than typical regional disease, and successful surgical cases may be overrepresented. Conversely, patients with slowly progressive or asymptomatic metastases may be underreported. The inclusion of cases over many decades introduces additional variability because diagnostic imaging, surgical techniques, histopathologic criteria, and molecular testing have changed substantially over time. Some older cases may not meet current diagnostic standards, whereas some modern cases may be detected earlier because of improved imaging. These factors should be considered when interpreting pooled estimates of metastatic site distribution and survival.

Despite these limitations, the study provides an updated pooled dataset of published case-report patients with MPA and offers several clinically relevant observations. First, MPA is frequently reported after a long interval and often in association with recurrent PA. Second, metastatic-site categories are heterogeneous and often non-mutually exclusive. Third, surgery is associated with better survival in univariable analyses, but this association is vulnerable to strong selection bias. Finally, the findings reinforce that MPA pathogenesis is likely multifactorial and requires multicenter registry and molecular studies rather than inference from retrospective case reports alone.

Future research should move beyond isolated case reports toward multicenter registries and collaborative studies. Because MPA is exceptionally rare, individual institutions are unlikely to accumulate enough cases for definitive analysis. Shared registries could enable standardized collection of clinical, radiologic, histologic, molecular, treatment, and outcome data. Central pathology review would help distinguish true MPA from carcinoma ex pleomorphic adenoma, myoepithelial carcinoma, and other mimics. Paired molecular analysis of primary PA, rPA, and metastatic lesions could clarify clonal evolution and identify alterations associated with metastatic competence. Such efforts may eventually allow for risk stratification of patients with PA or rPA and guide surveillance and treatment decisions more precisely.

## 5. Conclusions

MPA is a rare salivary gland neoplasm characterized by benign histologic morphology and metastatic behavior. In this pooled analysis of 122 published case-report patients, metastatic patterns were heterogeneous, follow-up and outcome reporting were incomplete, and survival analyses were limited by only 16 deaths. Surgical management was associated with improved survival in exploratory univariable analysis, but this association should not be interpreted as proof of an independent treatment effect. Surgery may be considered when technically feasible and clinically appropriate, particularly for selected patients with resectable disease.

MPA often occurs many years after initial PA treatment and is frequently associated with recurrent disease, although de novo and short-latency presentations may also occur. Long-term clinical awareness is particularly important after recurrent PA, multifocal disease, incomplete excision, or tumor spillage, but the current evidence does not define optimal surveillance intervals or imaging algorithms.

Available evidence supports a multifactorial model involving surgical factors, recurrence, tumor cell dissemination, molecular alterations, angiogenesis, and host microenvironmental conditions. Future multicenter studies with standardized reporting, centralized pathology review, formal reporting-completeness or risk-of-bias assessment, integrated molecular profiling, and longer follow-up are needed to identify patients at increased risk and to define optimal management strategies.

## Figures and Tables

**Table 1:** Characteristics of published case-report patients with metastasizing pleomorphic adenoma. Percentages use non-missing denominators; site categories are non-mutually exclusive.

Characteristic	Available *n*	Missing *n*	Result
Total published case-report patients	122	0	122
Sex: female	121	1	71/121 (58.7%)
Sex: male	121	1	50/121 (41.3%)
Primary site: parotid gland	122	0	92/122 (75.4%)
Primary site: submandibular gland	122	0	15/122 (12.3%)
Primary site: palate/soft palate	122	0	10/122 (8.2%)
Other primary sites:	122	0	5/122 (4.1%)
History of PA recurrence	116	6	80/116 (69.0%)
Multiple primary PA recurrences	116	6	46/116 (39.7%)
Number of primary PA recurrences	70	52	Median 2 (IQR 1–2; range 0–11)
Interval from PA to MPA	118	4	Median 12 y (IQR 7–21; range 0–69)
Metastatic site reported	120	2	Non-mutually exclusive categories
Bone/skeleton metastasis	120	2	42/120 (35.0%)
Lymph nodes/neck metastasis	120	2	42/120 (35.0%)
Lung/pulmonary metastasis	120	2	31/120 (25.8%)
Skin/soft tissue metastasis	120	2	17/120 (14.2%)
Kidney metastasis	120	2	10/120 (8.3%)
Liver metastasis	120	2	8/120 (6.7%)
CNS/intracranial	120	2	5/120 (4.2%)
Other visceral/mediastinal	120	2	5/120 (4.2%)
Multiple metastatic lesions	113	9	69/113 (61.1%)
MPA-directed surgery	98	24	86/98 (87.75%)
MPA recurrence after treatment	71	51	18/71 (25.4%)
Vital status: alive	85	37	69/85 (81.2%)
Vital status: dead	85	37	16/85 (18.8%)
Overall-survival analysis set	72	50	16 deaths

**Table 2:** Illustrative institutional cases. These cases are presented separately and were not included in the primary pooled case-report statistical analyses.

Case	Sex	Age at pPA Diagnosis	pPA Site	Recurrence	Age at MPA Diagnosis	MPA Site	Therapy	Outcome	Follow-Up
1	Female	30	SMG	Yes	35 years	Neck nodes	Surgery	Alive, no recurrence	52 months
2	Female	74	Soft palate	No	75 years	Neck nodes	Surgery	Alive, no recurrence	48 months
3	Female	19	Parotid gland	Yes	30 years	Neck nodes	Surgery	Alive, no recurrence	12 months
4	Female	16	Parotid gland	Yes	28 years	SCM + ST	Surgery	Alive, no recurrence	12 months

Abbreviations: MPA, metastasizing pleomorphic adenoma; pPA, primary pleomorphic adenoma; SCM + ST, sternocleidomastoid muscle and soft tissue; SMG, submandibular gland.

**Table 3:** Exploratory univariable Cox regression analyses for overall survival in the published case-report cohort. Hazard ratios are shown with 95% confidence intervals. No multivariable model was fitted because only 16 deaths were available; estimates are hypothesis-generating only.

Variables	*N*	Events	*p*-Value	HR	95.0% Confidence Interval for Hazard Ratio (HR)	
					Lower	Upper
**Age**	72	16	0.321	1.022	0.979	1.068
**Sex**	72	16	0.815	0.847	0.211	3.396
**Primary: salivary glands vs. other**	72	16	0.048	0.131	0.017	0.981
**Lung and/or bone metastases**	72	16	0.842	0.907	0.345	2.381
**Multiple lesions yes/no**	72	16	0.343	2.352	0.401	13.797
**Therapy: Surgery vs. other**	72	16	0.030	0.119	0.018	0.810

## Data Availability

The data supporting the findings of this study are available from the corresponding author upon reasonable request and are derived from the included publications and separately described institutional cases. The patient-level extraction table and statistical summary are provided as Supplementary Material.

## Supplementary Materials

The following supporting information can be downloaded at: https://www.mdpi.com/article/10.3390/cancers18142345/s1, Table S1: Patient-level extracted data for the 122 published case-report patients included in the primary pooled analysis. Table S2: Completed PRISMA 2020 checklist for this systematic review and pooled case-report analysis. The checklist has been moved to the Supplementary Material to improve main-text readability. Table S3: Database-specific search strategies and full-text exclusion reasons.
